# An In Vitro Method for Assessing the Efficacy of Antivenom Against Hemiscorpius lepturus Venom

**Published:** 2012-01-04

**Authors:** Mohammad Hassan Pipelzadeh, Mahsa Pipelzadeh

**Affiliations:** 1 Department of Pharmacology and Toxicology Research Centre, Ahvaz Jundishapur University of Medical Sciences, Ahvaz, IR Iran; 2 Department of Cellular and Molecular Biology, Tehran Medical Azad University, Tehran, IR Iran

**Keywords:** Hemiscorpion lepturus, Hemolysis, Antivenins

## Abstract

**Background:**

Hemolyis of red blood cells is a serious toxic effect commonly found among patients envenomed by Hemiscorpius lepturus scorpion.

**Objectives:**

The aim of the present study was to evlaute the efficay of the avaible polyvalent antivneom in preventing this phenomena.

**Materials and Methods:**

Using a red blood cell fragility test, the anti-hemolytic effectiveness of a new antivenom serum against Hemiscorpius lepturus venom was investigated. Hemolysis was measured using spectrophotometry.

**Results:**

Addition of venom (2, 10, 20, and 40 µg/ml) to 0.5 ml of 5% washed red blood cell suspension produced concentration-dependent hemolysis. Both the pre-incubation of red blood cell suspensions with various concentrations of antivenom (4%, 10%, and 20% v/v) and the co-administration of antivenom with 20 µg/ml venom resulted in concentration-dependent protection against hemolysis. Both the methods resulted in protection against hemolysis at the antivenom concentration of 20% (v/v). However, the inhibition of hemolysis after 24 h was found to be greater for red blood cell suspensions preincubated with antivenom (75% inhibition) than for red blood cell suspensions that were co-administered with antivenom and venom (50% inhibition).

**Conclusions:**

The results suggest that the antivenom against H. lepturus venom is useful in inhibiting hemolysis produced by the venom, but the duration of protection is relatively short and appropriate measures need to be taken, depending on the patients’ clinical progress, to re-administer the antivenom at intervals less than 8 h. This proposed treatment method merits further clinical assessment.

## 1. Background

Hemiscorpius lepturus (H. lepturus) is a dangerous scorpion found in Iran, Iraq, Yemen, and some parts of Africa (1). A limited number of clinical and experimental studies have reported that hemolysis is one of the most common symptoms that follow envenomation by this scorpion (2, 3). Serious concerns have been raised regarding the efficacy of the currently available polyvalent scorpion antivenom against this scorpion species, which is the most dangerous in Iran (2). Recently, a new polyvalent antivenom has been produced, which is claimed to contain antibodies against H. lepturus venom. However, the information provided by the manufacturer does not provide clear instructions to clinicians as to the optimal dose, or the most effective route or frequency of administration. It was, therefore, of interest to elucidate the efficacy and potency of this new antivenom against venom-induced hemolytic effects in an in vitro setting.

## 2. Objectives

For this purpose, red blood cell (RBC) fragility test was used as a model for the evaluation of the efficacy of the antivenom under different experimental conditions and durations of incubation.

## 3. Materials and Methods

### 3-1. Preparation of Venom Solution

Electroshock lyophilized H. lepturus venom was purchased from Razi Institute (Ahwaz, Iran). The venom was dissolved in normal saline solution to a final concentration of 1 mg/ml and immediately stored at 4°C until needed. 

### 3-2. Preparation of RBC Suspensions

Three 10-ml aliquots of heparinized human blood samples, donated by 3 volunteers, were used for preparing the RBC suspensions. A 2-ml aliquot of each sample was mixed with 6 ml of normal saline and centrifuged for 3 min at 3000 g. This process was repeated 3 times to ensure the removal of all blood plasma. The washed RBC suspension was finally diluted with normal saline to 5% concentration and stored at 4°C until assayed. 

### 3-3. Assessment of the Hemolytic Efficacy of the Venom

The aim of this series of experiments was to assess the hemolytic efficacy of the venom (Razi Institute, Karaj, Iran) and select a standard concentration for later experiments. For this purpose, triplicate Eppendorf® tubes for each of the 3 blood samples, containing 0.5 ml of RBC suspension and varying concentrations (2, 10, 20, and 40 µg/ml) of the venom, were incubated at 37°C. For the measurement of spontaneous hemolysis, control RBC samples were maintained under the same experimental conditions in the absence of venom. After varying periods (30 min and 2, 4, 8, 12, and 24 h) of incubation, the tubes were centrifuged at 14000 rpm for 3 min (Eppendorf centrifuge model 5410, Germany). A 100-µl sample of each supernatant was transferred to a 96-well plate and hemolysis was measured using a spectrophotometric plate reader (Sunrise plate reader; Tecan, Austria) by reading absorbance at 450 nm. 

### 3-4. Test of Antivenom Activity 

In this series of experiments, the effectiveness of antivenom in inhibiting hemolysis was assessed under 2 different experimental conditions: 

### 3-5. Test of Direct Neutralizing Capacity of the Antivenom 

In order to evaluate the efficacy of the antivenom in inhibiting hemolysis, 20 µg/ml of venom, an amount found to produce obvious hemolysis after 2 h of exposure, was mixed with 0%, 4%, 10%, or 20% (v/v) antivenom and allowed to stand for 5 min. Then, 0.5 ml of RBC suspension was added to the mixture. After centrifugation to pellet the intact RBCs, the optical density of supernatant samples was measured as described in section 3-3, following the same incubation periods. The degree of hemolysis, i.e., the change in optical density, was compared between the untreated and venom-treated RBC samples at the corresponding periods of incubation. 

### 3-6. Test of the Efficacy of Antivenom in the Presence of Washed RBCs 

In order to assess the effect of RBCs on the efficacy of the antivenom, i.e., its specificity, 0.5 ml of RBC suspension was mixed with different concentrations of antivenom (4%, 10%, or 20% v/v) and allowed to stand for 5 min. Then, 20 µg/ml of the venom was added to each sample and incubated as described in section 3-3. Hemolysis was measured and compared with both an untreated negative control and a positive control containing 20 µg/ml of venom only. In order to assess any possible hemolytic activity of the antivenom, a separate series of experiments was conducted: similar concentrations (4%, 10%, or 20% v/v) of antivenom were mixed with 0.5 ml of RBC suspension and incubated as described in section 3-3. The resultant changes in hemolysis were compared with an untreated control. 

### 3-7. Statistical Analysis 

The results were expressed as mean ± SEM. The significance of differences between the means for different treatment groups was assessed using ANOVA followed by Duncan,s test for multiple comparisons. Values of P < 0.05 were considered significant.

## 4. Results

### 4-1. Hemolytic Effect of H. lepturus Venom on RBCs 

**Figure 1:**
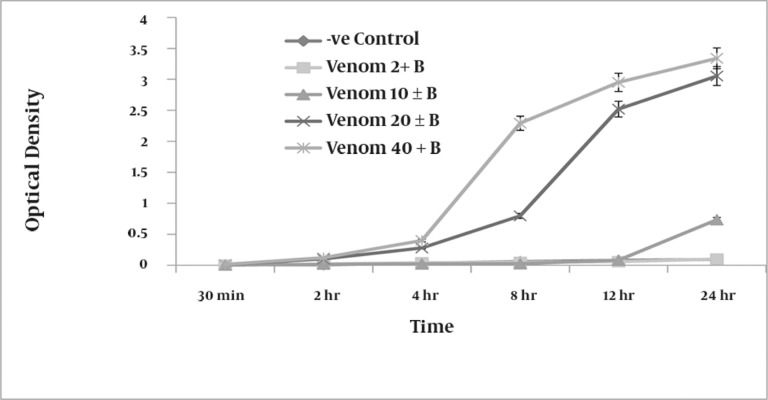
A graphic representation of the mean ± SEM (n = 9) of the extent of haemolysis produced by various concentrations of H. lepturus venom (2, 10, 20 and 40 g/ml of 5 % washed RBC) following progressive incubation periods ranging from 30 min to 24 hours, B = washed RBC.

Compared with negative controls, which were not exposed to venom, RBCs treated with venom showed concentration-dependent hemolysis. At a concentration of 20 µg/ml, the venom produced measurable (optical density of 0.122) hemolysis of the RBC suspension after only 2 h of incubation. Increased concentrations of venom produced an increase in the degree of hemolysis (Figure 1). At 40 µg/ml, the venom caused complete lysis of RBCs after 24 h and produced a clear red solution devoid of intact RBCs (optical density of 3.5). 

### 4-2. Direct Neutralizing Efficacy of the Antivenom

**Figure 2:**
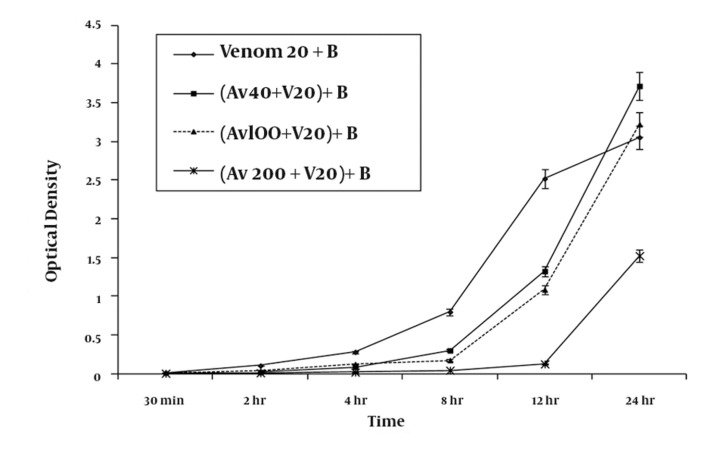
A graphic representation of the mean ± SEM of the efficacy of various concentrations of antivenom (Av) in preventing haemolysis produced by 20 ug/ ml H. lepturus venom (V) on 5 % washed RBC (B) taken from three healthy volunteers following premixing of the venom with antivenom serum (n = 9).

Co-administration of antivenom with 20 μg/ml venom inhibited RBC hemolysis in a concentration-dependent manner (Figure 2). At the highest tested concentration of antivenom (20% v/v), protection against hemolysis was sustained for up to 12 h, with a degree of hemolysis similar to that measured for negative-control RBC suspensions not exposed to venom (Figure 2). However, after 24 h of incubation, the extent of hemolysis was 50% (optical density of 1.524 ± 0.07) of that produced by venom-treated standard samples (optical density of 3.054 ± 0.15), indicating a time-dependent loss of antivenom protection.

### 4-3. Effect of RBCs on the Efficacy of the Antivenom

**Figure 3:**
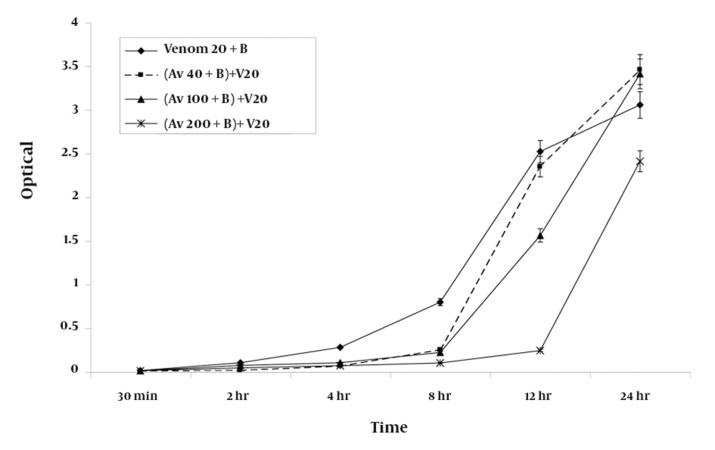
A graphic representation of the mean ± SEM of the efficacy of various concentrations of antivenom (Av) in preventing haemolysis produced by 20 ug/ ml of H. lepturus venom (V) on 5 % washed RBC (B) following premixing of the venom with the red blood cells suspension (n = 9).

Premixing antivenom with RBC suspensions produced inhibition of hemolysis at the 8-h time point; this was similar to the inhibition that was observed after co-administration of the antivenom with venom (Figure 3).

The extent of inhibition of hemolysis in RBC suspensions premixed with 20% (v/v) antivenom was found to be 75% (2.409 ± 0.11) of that measured for standard positive control containing 20 μg/ml of venom (3.054 ± 0.15) after 24 h of incubation. Incubation of antivenom at various concentrations with the RBC suspension over the period of study did not produce significant degree of hemolysis from those measured for negative control.

## 5. Discussion

In this study, the use of human RBC suspensions in conjunction with a spectrophotometric reading method was found to be a useful, simple, and rapid test for the detection of hemolysis in an in vitro setting. The findings of these experiments showed that the venom from H. lepturus is a potent toxic agent with a long acting hemolytic action. At 40 μg/ml concentration, H. lepturus venom produced complete hemolysis of a 5% washed RBC suspension within 24 h. Furthermore, the antivenom protected RBCs from hemolysis for up to 12 h when co-administered with the venom, and for up to 8 h when premixed with RBC suspensions before the addition of venom. These findings suggest that although the antivenom is a useful agent in the treatment of envenomation, it has limited efficacy and specificity. Our testing methodology was found to be simpler and more rapid than the methodologies used by Seibert et al., who used an RBC fragility test in hemolymph samples taken from envenomed animals by Lonomia obliqua caterpillars (2). Our proposed method is fast and simple, and in addition, it can be used to assess the hemolytic potency of venom at different time points and to assess the effectiveness of antivenom in inhibiting the hemolysis produced by various concentrations of the venom. The results of such assessments can give useful guidelines for improvement of treatment. Previous clinical (3),(4) and experimental (5),(6) studies have shown that H. lepturus venom acts as a cytotoxic agent and produces serious cutaneous inflammatory reactions and necrosis at the site of the sting, similar to the lesions produced by the brown recluse spider (Loxosceles reclusa) (1). In addition, H. lepturus venom has nephrotoxic properties, which may represent a secondary effect of its hemolytic action. Classically, the quantity of antivenom necessary to neutralize 1 mg of venom is considered the treatment dose (7). However, in practice, the efficacy of the antivenom is usually lower than stated, and some researchers advocate the administration of 5 to 10 times the stated dose for the treatment of envenomed patients (8). In our study, the concentration of antivenom needed to produce sufficient protection against 20 μg/ml venom for up to 8 h was 200 μl/ml. Extrapolation from these data suggests that the commercially available 5-ml (5000 μl; half of the classically recommended dose) injection form of the antivenom is capable of neutralizing 500 μg of venom. The amount of the venom extractable from individual scorpions following electrical stimulation has been reported to be 130 ± 80 μg (8); on the basis of the findings of this study, a single ampoule of the antivenom needed to provide protection against hemolysis for up to 8 h was approximately 2 to 3 times the calculated dose. However, it should be considered that in this study, premixing antivenom with RBC suspensions reduced the efficacy of the antivenom by approximately 30%. In summary, the findings of this study suggest that the antivenom has a limited period of efficacy lasting up to 12 h, and it is expected that under in vivo conditions, this efficacy is likely to be further reduced. What is the relevance of these findings to clinical practice? Although extrapolation of in vitro data to clinical settings should be performed with caution, the findings from this study nevertheless demonstrated that one major action of H. lepturus venom can easily and accurately be quantified at multiple time points, and can be used to monitor hemolysis in the envenomed patients. Furthermore, although the commercially available antivenom has protective effects against the hemolytic actions of H. lepturus venom, it has limited efficacy and specificity. It seems likely from the findings of this study that repeated antivenom dosing, rather than the currently used routine single intramuscular injection, in conjunction with close clinical observation would benefit envenomed patients irrespective of their age. Further independent clinical studies would be required to assess this suggested treatment method.
